# Epstein–Barr virus-mediated transformation of B cells induces global chromatin changes independent to the acquisition of proliferation

**DOI:** 10.1093/nar/gkt886

**Published:** 2013-10-03

**Authors:** Henar Hernando, Abul B. M. M. K. Islam, Javier Rodríguez-Ubreva, Ignasi Forné, Laura Ciudad, Axel Imhof, Claire Shannon-Lowe, Esteban Ballestar

**Affiliations:** ^1^Chromatin and Disease Group, Cancer Epigenetics and Biology Programme (PEBC), Bellvitge Biomedical Research Institute (IDIBELL), Avda. Gran Via 199-203, 08908 L'Hospitalet de Llobregat, Barcelona, Spain, ^2^Department of Experimental and Health Sciences, Barcelona Biomedical Research Park, Universitat Pompeu Fabra (UPF), 08003 Barcelona, Spain, ^3^Department of Genetic Engineering and Biotechnology, University of Dhaka, Dhaka 1000, Bangladesh, ^4^Center for Integrated Protein Science and Adolf-Butenandt Institute, Ludwig Maximilians University of Munich, 80336 Munich, Germany and ^5^CR-UK Institute for Cancer Studies, University of Birmingham, Birmingham B15 2TT, UK

## Abstract

Epstein–Barr virus (EBV) infects and transforms human primary B cells inducing indefinite proliferation. To investigate the potential participation of chromatin mechanisms during the EBV-mediated transformation of resting B cells we performed an analysis of global changes in histone modifications. We observed a remarkable decrease and redistribution of heterochromatin marks including H4K20me3, H3K27me3 and H3K9me3. Loss of H4K20me3 and H3K9me3 occurred at constitutive heterochromatin repeats. For H3K27me3 and H3K9me3, comparison of ChIP-seq data revealed a decrease in these marks in thousands of genes, including clusters of *HOX* and *ZNF* genes, respectively. Moreover, DNase-seq data comparison between resting and EBV-transformed B cells revealed increased endonuclease accessibility in thousands of genomic sites. We observed that both loss of H3K27me3 and increased accessibility are associated with transcriptional activation. These changes only occurred in B cells transformed with EBV and not in those stimulated to proliferate with CD40L/IL-4, despite their similarities in the cell pathways involved and proliferation rates. In fact, B cells infected with EBNA-2 deficient EBV, which have much lower proliferation rates, displayed similar decreases for heterochromatic histone marks. Our study describes a novel phenomenon related to transformation of B cells, and highlights its independence of the pure acquisition of proliferation.

## INTRODUCTION

Quiescent differentiated B lymphocytes, like many other types of differentiated cells, have a relatively restricted transcriptome generated through the differentiation process. This restriction of the transcription program in differentiated resting cells is associated with the epigenetic silencing of the genome, which forms blocks of facultative heterochromatin (1). Histone modifications associated with facultative heterochromatin include H3 trimethylation at Lys 27 (H3K27me3), H3 di- and trimethylation at Lys 9 (H3K9me2/H3K9me3) and H3 and H4 hypoacetylation (2). This type of heterochromatin coexists with constitutive heterochromatin, another class of silent chromatin quite different from facultative heterochromatin. Constitutive heterochromatin comprises gene-poor DNA containing highly repetitive sequences, is characterized by H4K20me3 as well as H3 and H4 hypoacetylation (2), and is independent of cell differentiation and proliferation status, as well as the level of transcriptional activity. Both types of heterochromatin are highly condensed and are less accessible to endonucleases than euchromatin, characteristic of transcriptionally active regions. In fact, there is a general notion that certain histone modifications are associated with a particular chromatin structure. One of such examples is H3K27me3 that is generally associated with condensed facultative heterochromatin, less accessible to nucleases, and both associated with low levels of expression.

The B cell compartment in peripheral blood consists of quiescent naïve and memory cells that can be activated and driven into proliferation in response to encountering an antigen. This phenomenon can be replicated *in vitro* through co-stimulation with IL-4 and CD40L that leads to a finite proliferative lifespan. In contrast, experimental infection of resting B cells (RBLs) with Epstein–Barr virus (EBV) results in the establishment of lymphoblastoid cell lines (LCLs) with indefinite proliferation (3). Transition from quiescence to proliferation involves major changes in gene expression, nuclear reorganization, and requires the participation of various pathways, including cell signaling and cell cycle factors and elements of the epigenetics and chromatin machinery. Infection of B cells with EBV, which is highly prevalent in humans, is an excellent model not only for investigating the molecular mechanisms associated with the transition from quiescence to proliferation but also for understanding those related with growth transformation. In fact, EBV-associated changes in B cells are relevant to the development and progression of lymphomas (4–6), lymphoproliferative disorders in immune-suppressed individuals, and autoimmune disorders like rheumatoid arthritis, systemic lupus erythematosus and multiple sclerosis (7). *In vitro*, EBV efficiently immortalizes primary RBLs, converting them into permanently growing LCLs. *In vitro* infection results in the activation of a specific viral gene expression program that involves expression of six nuclear antigens (EBNA-1, -2, 3A, -3B, -3C and -LP) and three membrane proteins (LMP-1, -2A and -2B). Five of these proteins are essential for transformation. For instance, LMP-1 is required for the establishment of cell transformation *in vitro* (8) and is required for continuous proliferation (9). Infection of B cells with EBV is similar to the physiological stimulation with CD40L plus IL-4 (3), T cell-derived mitogens, and in both cases involves the activation of the NF-kB pathway.

EBV-mediated transformation of RBLs to proliferating lymphoblasts involves changes to the expression profile and is likely to result in reorganization of the histone modification profiles. In this process, some of the changes in the histone profiles occur through the direct recruitment of histone modification enzymes by viral transcription factors like EBNA-2 (10,11) and are specific to the activity of EBV proteins. Others perhaps occur through indirect mechanisms or even as a result of the acquisition of the proliferation. In this process it is essential to identify which of these effects are EBV-specific and which could be classified as general changes associated with the transformation from quiescent to proliferative B cells, also as initial steps in lymphomagenesis.

In this study, we have investigated global changes in histone modifications as well as accessibility to endonucleases during the EBV-mediated transformation of RBLs to proliferative lymphoblasts. In this transition, we have observed a significant reduction of heterochromatic histone modifications like H3K27me3, H3K9me3 and H4K20me3 over time, whereas no significant changes in H3K4me3 and other euchromatin-specific marks were observed. A decrease in heterochromatin histone marks occurred at repetitive elements for H4K20me3 and at unique genomic sites for H3K9me3 and H3K27me3, particularly those genomic regions highly enriched in these marks, including ZNF and HOX gene clusters, respectively. We have also observed that infection with EBV results in increased endonuclease accessibility at thousands of genomic sites. Both decrease in heterochromatic histone modifications and increased accessibility to endonucleases in proliferating versus RBLs are associated with increased transcriptional activity. Changes in heterochromatin histone modifications are specific to EBV-mediated transformation, whereas cytokine stimulated activation and proliferation of B cells do not result in significant decrease of these marks. In addition, B cells infected with EBV particles deficient for EBNA-2 that result in much lower proliferation rates also undergo decrease in these marks. Our study describes the existence of global changes in histone heterochromatin marks and chromatin compaction in EBV-mediated transformation of B cells. The comparison with IL-4/CD40L-stimulated cells and B cells infected with mutant EBV particles highlights the independence of this process with the acquisition of proliferative properties providing novel clues of the existence of specific epigenetic alterations in the growth transformation of B cells.

## MATERIALS AND METHODS

### Ethics statement

Human blood samples used in this study came from anonymous blood donors and were obtained from the Catalan blood donation center (Banc de Sang i Teixits). The blood anonymous donors received both verbal and written information about the possibility that their blood was used for research purposes, their questions were then answered and they signed a consent form at the Banc de Teixits. The Banc de Teixits follows the principles set out in the WMA Declaration of Helsinki. The protocol used to transform B cells from these anonymous donors with EBV was approved by the Committee of Biosecurity of IDIBELL (CBS) on 5 May 2011 and the Ethics Committee of the University Hospital of Bellvitge (CEIC) on 28 May 2011.

### B cell isolation and transformation with EBV

Viable peripheral blood mononuclear cells (PBMCs) were isolated by LymphoprepTM density gradient centrifugation from buffy-coats from anonymous blood donors. RBLs were isolated by positive selection using CD19 MicroBeads (Miltenyi Biotec), or by depletion using a B Cell Isolation Kit (Miltenyi Biotec). Isolated B cells were immortalized with the supernatant of the 2089 EBV variant made from 293 cells carrying a recombinant B95.8 EBV genome (12). Preparations of the 2089 recombinant wild type EBV with a GFP insert or viruses deleted for LMP-1 and EBNA-2 (13) were made from 293 cells carrying the recombinant B95.8 EBV genomes, and transfected with 0.5 µg BZLF1 (p509) + 0.5 µg gp110 (pRA). We purified the encapsidated and enveloped virus particles from culture supernatants by centrifugation on an Optiprep (Axis Shield) self-generated gradient and then quantitated by a qPCR assay amplifying a single copy gene, BALF5, using Namalwa cell line (two integrated copies of EBV) as the standard. The multiplicity of infection (MOI) was defined as the number of EBV genomes in the purified virus preparations divided by the number of target cells in culture. We then incubated freshly isolated primary B cells with purified virus supernatants at a 100 MOI for 3 h at 37°C. The virus-loaded B cells were then plated according with the experiment.

### B cell activation

Isolated B cells were cultured 5 × 10^6^/3 ml per well of a 6-well plate with 50 ng/ml CD40L (Enzo Life Sciences) and 50 ng/ml IL-4 (Gentaur) and the B cell blasts were split at a 1:2 ratio once a week. The percentages of activated and proliferating B cells were detected by CD86 expression measured by flow cytometry and tritiated thymidine incorporation, respectively.

### LMP-1 lentiviral infection

B cells activated during 4 days were infected with the GFP-lentiviral vector expressing LMP-1, then added appropriate numbers of cells to each plate, i.e. 1 × 10^5^ B cells to 1 well of a 24-well plate. We then added 2 ml of lentivirus supernatant to these cells and incubated at 37°C for 30 min. Then the cells were centrifuged at 30°C for 2 h at 2200 rpm. After 18 h of incubation the medium was replaced with fresh medium. The efficiency of the infection was measured by the GFP expression in the infected cells using FACS.

### Quantification of cell proliferation by tritiated thymidine incorporation

The isolated CD19 positive cells EBV-infected or CD40L/IL-4-stimulated were plated in 24-well plates in a concentration of 250 000 cells/well in 1 ml of RPMI 10% FBS 1% antibiotic/antimycotic solution. We exposed cells to 0.4 uCi/ml of [3H]thymidine for 16 h. Then cells were washed 3 times with PBS 1× and fixed with 1 ml of 5% trichloroacetic acid in PBS for 15 min at 4°C. The fixative solution was removed and cells were washed three times with 1 ml of methanol. After the total elimination of methanol by air-drying cells were dissolved with 0.5 ml of NaOH 0.1 M and 1% SDS and transferred to the scintillation vial with 2.25 ml of scintillation fluid and mixed by vortex. The CPM was measured in the liquid scintillation counter.

### Quantification of cell activation by CD86 expression

EBV-infected or CD40L/IL-4-activated B cells were harvested and stained for CD86 expression with an anti-human CD86 FITC-conjugated (EuroBioSciences) at 1:100 concentration. CD86 levels were determined by flow cytometry (Gallios™ Flow Cytometer—Beckman Coulter).

### Antibodies

The following rabbit polyclonal antibodies were used: H3K4me3 (Millipore CS200580), H3K9me3 (Millipore 07-442 (branched) / Abcam 8898), H3K27me3 (Millipore 07-449 for western blot; Diagenode cs-069-100 for ChIP assays), H4K20me1 (Abcam ab9051), H4K20me2 (Millipore 07-1584) and H4K20me3 (Millipore 07-463). We also used antibodies against the monoacetylated forms at specific residues of histone H4 (K5, Millipore 07-327; K8, Abcam ab15823; K12, Abcam ab1761; and K16, Millipore 07-329). Control antibodies for histone H3 (Abcam ab1791-100) and H4 (Abcam ab10158) were also used.

### Western blotting

Histones were separated on 15% SDS-PAGE gel and blotted onto a polyvinylidene difluoride membrane of 0.22 µm pore size (Immobilon PSQ, Millipore). The membrane was blocked in 5% milk PBS-T (phosphate-buffered saline with 0.1% Tween-20) and immunoprobed with antibodies raised against different peptides containing different histone modifications, as described above. The secondary antibodies used were goat anti-rabbit conjugated to horseradish peroxidase (HRP) (1:3000) (Amersham) and sheep anti-mouse-HRP (1:5000). Experiments were performed in triplicate. Bands were quantitated by direct scanning of the western blot films with a HP scanjet 4890 and processed with ImageJ software.

### Immunofluorescence

Cells were grown on coverslips in P60 dishes, fixed in 100% methanol, and stained as described previously (14). Fluorochromes were DAPI and Alexa 488. Confocal optical sections were obtained using a Leica TCS SP5 Spectral confocal microscope (Leica Microsystems) equipped with krypton and argon lasers. Operation was done at a constant temperature of 22°C. Images were acquired with Leica Application Suite Advanced Fluorescence (LAS AF) software and processed with FIJI software using the following pluggins: Feature Extraction-FeatureJ Laplacian (Copyright (C) Erik Meijering) for H4K20me3 and Plot profile feature for H3K27me3 and H3K4me3.

### Mass spectrometry

Histone bands were excised from SDS-PAGE gels and digested in-gel as described earlier in Jasencakova *et al.* (15). Briefly, histones were destained and propionylated before trypsin digestion was performed. After digestion, peptides were desalted offline using Carbon carbon TopTips (TT1CAR, Glygen) and reconstituted in 0.1% TFA.

The peptides were injected in an Ultimate 3000 HPLC system (LC Packings Dionex and separated with a gradient from 5% to 60% acetonitrile in 0.1% formic acid over 40 min at 300 nl/min on a C18 analytical column (75 µm ID homepacked with ReproSil-Pur C18-AQ 2.4 µm from Dr Maisch). The effluent from the HPLC was directly electrosprayed into the LTQ Orbitrap mass spectrometer (Thermo Fisher Scientific). The MS instrument was operated in the data-dependent mode to automatically switch between full scan MS and MS/MS acquisition. Survey full scan MS spectra (m/z 250 – 2000) were acquired in the Orbitrap with resolution *R* = 60 000 at m/z 400. The six most intense peptide ions with charge states between two and five were sequentially isolated (window = 2.0 m/z) to a target value of 10 000 and fragmented in the linear ion trap by collision-induced dissociation (CID). Fragment ion spectra were recorded in the Orbitrap part of the instrument. For all measurements with the Orbitrap detector, three lock-mass ions from ambient air (m/z = 371.10123, 445.12002, 519.13882) were used for internal calibration. Typical mass spectrometric conditions were: spray voltage 1.4 kV; no sheath and auxiliary gas flow; heated capillary temperature 200°C; normalized collision energy 35% for CID in linear ion trap. An activation *q* = 0.25 and activation time of 30 ms were used. Peptides were quantified using the peak area from the corresponding extracted ion chromatograms (XICs, ±10 ppm).

Peptides were quantified using the peak area from the corresponding XICs (±10 ppm). To avoid differences originating from the amount of material, digestion efficiency and spray fluctuation during the LC-MS/MS analysis, peptides were normalized to the peak area of the corresponding heavy labeled peptides, spiked into the sample prior to trypsin digestion.

### ChIP assays

To isolate genomic sites associated with specific histone modifications standard chromatin immunoprecipitation (ChIP) assays were performed as previously described (16). Commercial antibodies for H3K9Me3, H3K27me3, H3K4me3 and H4K20me3, and H3 and H4 were the same as those used for western blot analysis. Immunoprecipitated material was used for analyses of specific sequences by quantitative real time PCR (see primers sequences in Supplementary Table S1) as well as for hybridization on Southern blot assays.

### Micrococcal nuclease and DNase I digestions and Southern blot analysis

Resting and lymphoblastoid B cells were resuspended in equal volumes of PBS and cell lysis buffer (0.65 M sucrose; 20 mM Tris, pH 8; 10 mM MgCl_2_; 2% Triton X-100) and incubated on ice for 15 min. Cells were centrifuged at 1500 rpm for 5 min, and the nuclear pellet was resuspended in 1 ml of NT buffer (50 mM Tris pH 7,4; 100 mM NaCl; 5 mM MgCl_2_; 5 mM CaCl_2_; 1% NP-40; 1% Triton X-100). For both MNase and DNase I digestions we followed the manufacturers’ instructions and stopped the reactions at different times (30 s, 1, 2, 4, 8, 16 and 32 min). MNase was used at 0.01 unit/µl and DNase I at 1 unit/µl. Products of digestion products were separated on 2% agarose gels. These gels were used in two alternative experiments. In some cases, we performed quantitative PCR to analyse fragments in the ≥5000 bp range isolated from agarose gels (see primers sequences on Supplementary Table S1). In other cases, we performed Southern blot, to which end, DNA samples were transferred to Hybond-N+ membranes and ChIP products were labeled with [α32P]dCTP with the Ready-to-Go Labelling Kit (Amersham) and used as a probe. Experiments were performed with three biological replicates.

### Bioinformatic analysis

#### Analysis of expression data

Normalized expression data were extracted from the GEO database accession GSE30916 (17). Differential expression (log_2_) was calculated from the average expression of LCLs versus the average expression of primary B cells, corresponding to matching paired samples from a total of five individuals in duplicate. Probes were annotated to EnsEMBL (version-65) genes (18) using Biomart (www.biomart.org) database. In case of duplicate IDs we used the highest absolute expression value for maximization.

#### ChIP-seq and DNase-seq data sources and data processing

Sources of ChIP-seq and DNAse-seq data are detailed in Supplementary Table S2. We downloaded either aligned BED format files or BAM format files from public databases. BAM files were converted to BED format files using BETools (bamToBed function) (19). When there were more than one replicate, they were concatenated to obtain wider coverage and greater sequence depth.

#### Analysis of differential association of histone modifications

Differential association (increased or decreased genomic location) of H3K4me3, H3K27me3 and H3K9me3 was analysed using the MACS program (version 2.0.9; macs2diff function) (20) using the parameter settings: -g hs –nomodel –shiftsize = 75 –bdg -a 4 -q 0.005. Therefore, *q*-value cutoff was established at 0.005. MACS's macs2diff function establishes differential regions by comparing two treatment files corresponding to two conditions (in this case, LCLs and RBLs), and comparing each of them against corresponding control ‘Input’ files. This comparison generates an output containing a list of ‘differentially bound locations’ with a ‘differential score’ (-Log_10_ *q*-value) where positive number means that the signal in LCLs is higher than in RBLs, and negative number means the opposite. Higher scores indicate that the difference between the two cell types is larger. Both unique and consistently found differentially bound locations were considered for further analysis, for example correlation with expression. However, differential locations with score (-Log_10_ *q*-value) exactly zero were removed from analysis. When more than one replicate of the aligned file were present, they were merged to ensure wider coverage and greater sequencing depth. Significantly differentially bound locations were annotated to the closest EnsEMBL (version-65) transcripts and genes (18) using BEDTools (closestBed function) (19). We calculated Pearson’s correlation coefficient (PCC) between the differentially bound location scores, in which negative and positive values, respectively, indicate decreased and increased location scores in LCLs/RBLs comparisons, and the differentially expressed gene expression values (log_2_-fold change; microarray data from GEO GSE30916).

#### Analysis of differential accessibility at genome-wide level

DNase-seq (increase or decrease in open-chromatin genomic location scores) in GM12878/CD20 was analysed using the SICER program (version 1.1; SICER-rb-df function; window = 200, gap = 0, FDR = 0.01) (21). When more than one replicate of the aligned file was present, they were merged to ensure more coverage and sequencing depth. Identified significantly differentially DNase-seq locations were annotated to the closest EnsEMBL (version-65) transcripts and genes (18) using BEDTools (closestBed function) (19).

We calculated PCC between the differential change in open-chromatin location (negative and positive values, respectively, indicate decreased and increased location scores for the LCL/RBL comparison) and differentially expressed gene expression values (log_2_-fold change; microarray data from GEO GSE30916; LCL/RBL).

Genomic location overlap analysis: Overlap of decreased H3K9me3 and H3K27me3 locations with increased Dnase-seq open-chromatin locations was obtained using BEDTools (intersectBed function) (19). In this case, the overlap has a threshold of at least 1 nt.

#### GO functional enrichment analysis

Functional annotation of differentially expressed genes is based on Gene Ontology (GO) [(22); http://www.geneontology.org], as extracted from EnsEMBL (version 65) (18). Accordingly, all genes are classified into three ontology categories: (i) biological process (BP); (ii) cellular component (CC); and (iii) molecular function (MF) and pathways when possible. We took only the GO/pathway categories, which have at least 10 genes annotated. We used Gitools for enrichment analysis and heat-map generation ((23); www.gitools.org). Resulting *P*-values were adjusted for multiple hypothesis testing using Benjamini and Hochberg’s False Discovery Rate (FDR) method (24).

## RESULTS

### Loss and redistribution of H3K27me3, H3K9me3 and H4K20me3 during EBV-mediated transformation of B cells

**Figure 1.**
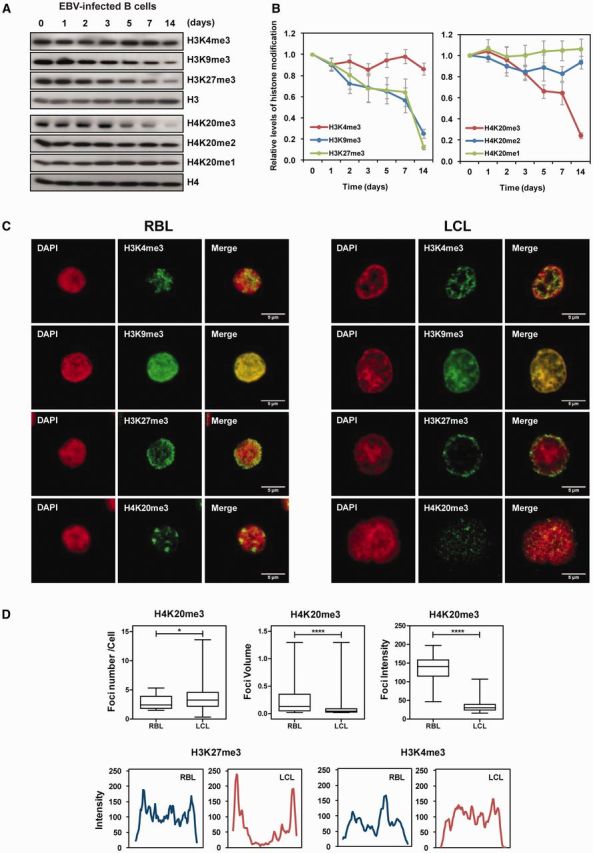
Global analysis of histone modifications during EBV-mediated transformation from resting to proliferative B cells (**A**) Analysis by western blot of a time course experiment with antibodies against H3K4me3, H3K9me3, H3K27me3 and total H3; H4K20me3, H4K20me2, H4K20me1 and total H4. (**B**) Densitometric quantitation of western blot data. The average of three independent experiments is represented. Data are normalized against the amount of total histone. (**C**) Analysis of H3K4me3, H3K9me3, H3K27me3 and H4K20me3 distribution in resting and proliferative B cells by immunofluorescence microscopy. Bars, 5 µm. (**D**) Quantitative analysis of immunofluorescence data. For H4K20me3, box and whisker plots (top graphs) show the number of foci per cell, foci volume and intensity. The bottom and top of each box are the 25th and 75th percentile and the bar near the middle the 50th percentile (the median). H3K27me3 and H3K4me3 cell diameter plots are represented at the bottom. Numbers are the values of the integrated density of the total fluorescent emission per nuclear area of H3 and H4 signal. 50 nuclei were analysed in each case. Values are shown as averages (±S.D.) in arbitrary units.

EBV can very efficiently induce the activation and continuous proliferation of RBLs. This involves a dramatic change in the biology of the cell, including an increase in the nucleus size (Supplementary Figure S1) and expression changes affecting thousands of genes. To investigate the potential involvement of changes in histone modifications in association with transformation of resting to proliferating B cells, we first performed a time course analysis of global levels of histone modifications by western blot. We infected RBLs with EBV and analysed the levels of H3K4me3, H3K9me3 and H3K27me3, as well as the mono-, di- and trimethylated forms of K20 of histone H4 at different time points, up to 1 and 2 weeks after infection when cells have become LCLs (Figure 1A). We also screened different histone H4 monoacetylated forms (H4K5Ac, H4K8Ac, H4K12Ac and H4K16Ac) (Supplementary Figure S2). Our analysis included control antibodies for the C-termini of histones H3 and H4 and quantitation of three biological replicates. To ensure the sensitivity of our system we used a strain of EBV that infects B cells very efficiently (12) (around 90% of B cells express the EBV transcription factor EBNA-2 at 24 h) allowing inspection of changes in histone modifications at early time points after infection (24, 48 and 72 h), before cells display active proliferation.

This analysis revealed a significant and progressive decrease in histone modifications associated with repression, specifically H3K27me3, H3K9me3 and H4K20me3 (Figure 1A). In contrast, no differences were observed for H3K4me3, an archetypical marker of gene activation, and for the monoacetylated forms of histone H4 (Supplementary Figure S2). Scanning and quantitation of the three independent experiments confirmed all these observations (Figure 1B and Supplementary Figure S2). A decrease in histone modifications associated with repression and condensed chromatin is compatible with the type of nuclear and expression changes associated with B cell transformation.

We also compared the nuclear distribution of these histone modifications in RBLs and LCLs. H4K20me3 staining in RBLs is characterized by a speckled pattern with large foci. In LCLs, the size and number of foci became smaller and the pattern observed was more diffuse (Figure 1C and D). H3K27me3 showed a very different pattern, characterized in RBLs by a more diffuse distribution with an accumulation of the signal toward the periphery of nucleus. In LCLs, H3K27me3 showed a sharper distribution with a less intense staining of the central part of the nucleus and a stronger staining at the periphery when compared to RBLs (Figure 1C and D). In the case of H3K9me3, we did not observe a clear change of distribution of the mark when comparing RBLs and LCLs. We did not observe any changes for H3K4me3 either. In summary, our results not only revealed the decrease in the global levels of heterochromatic histone marks but also the acquisition of profound changes in their nuclear distribution.

Changes in these histone modifications could be due to several causes, including mistargeting and aberrant overexpression or downregulation of the enzymes responsible for the deposition of these marks. Interestingly, the H3K27me3 demethylase KDM6B (JMJD3) has recently been described as being induced by EBV (25). Also, EZH2, the major H3K27 methyltransferase, is mutated in certain B cell lymphomas (26). We therefore checked the expression levels of these genes as well as all those encoding for enzymes involved in the incorporation/elimination of H3K27me3, H3K9me3 and H4K20me3 in an expression dataset corresponding to both resting and proliferating B cells (GSE30916) (17) (Supplementary Figure S3). Interestingly, some of the genes encoding for these enzymes like *EZH2*, *SUV39H1* and *SUV39H1* (H3K9 methyltransferase), and *SUV420H1* and *SUV420H2* (H4K20 methyltransferase) displayed expression changes compatible with the observed changes of their Lys substrates. However, changes in other enzymes also targeting these heterochromatin marks did not directly correlate with the observed differences between RBLs and LCLs (Supplementary Figure S3).

### Identification of genomic sites undergoing decreases in H4K20me3, H3K27me3 and H3K9me3 during EBV-mediated transformation of B cells

**Figure 2.**
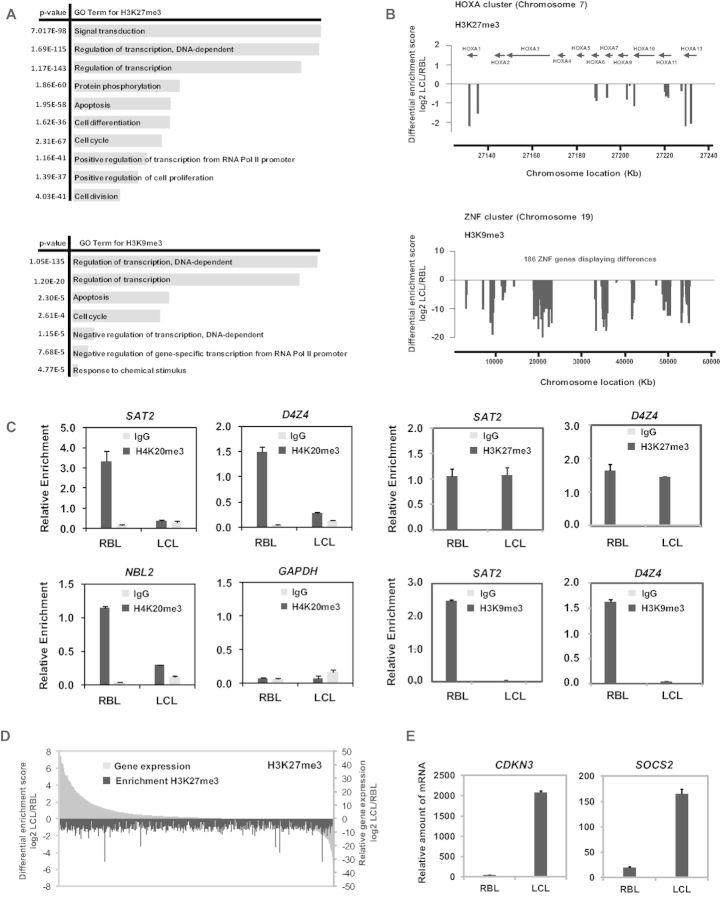
Changes in heterochromatic histone modifications in specific sequences. (**A**) Gene ontology biological process analysis of the sites with differential association for H3K27me3 and H3K9me3. The bar length is proportional to the number of genes for a given category. (**B**) Examples of gene clusters that exhibit significant decreases during the conversion of RBLs to LCLs. The HOXA cluster (at chromosome 7) for H3K27me3 and ZNF cluster (at chromosome 19) for H3K9me3. The *y*-axis shows the differential association of these two histone marks between RBLs and LCLs. (**C**) Quantitative ChIP assays showing changes in H4K20me3, H3K27me3 and H3K9me3 between RBLs and LCLs at repetitive sequences (SAT2, D4Z4, NBL2) characterized by constitutive heterochromatin. GAPDH is used as a control gene. (**D**) Analysis of expression changes for genomic sites undergoing a decrease in H3K27me3. All genes displaying a decrease in H3K27me3 enrichment in LCLs with respect to RBLs (blue bars) are shown including their corresponding change in expression (red bars), both in log2 scale. (**E**) Quantitative RT-PCR analysis showing a couple of example of genes displaying decreased H3K27me3 in LCls with respect to RBLs and increased expression in LCLs. Data are normalized against the RPL38 gene.

We then explored the genomic sites associated with the losses of H3K27me3, H3K9me3 and H4K20me3 during the EBV-mediated transformation of RBLs. H3K27me3 and H3K9me3 are present in facultative heterochromatin which covers a wide range of genomic sites. To investigate the genomic sites where H3K27me3 and H3K9me3 undergo changes between RBLs and LCLs, we compared ChIP-seq data between resting and proliferating B cells (GSE19465 for RBLs, ENCODE data for GM12878 for LCLs, described in detail in Materials and Methods section). We also analysed H3K4me3, for which we had not observed overall changes in our initial screening. This analysis revealed that a large number of genomic sites have a reduced association with H3K27me3 and H3K9me3. For H3K27me3, 5724 unique genomic sites featured a significant decrease in association from RBLs to LCLs (*q* < 0.005) (Supplementary Table S3). In the case of H3K9me3, 5298 unique sites underwent a significant decrease from RBLs to LCLs (*q* < 0.005) (Supplementary Table S3). Under the same conditions, only 275 peaks were significantly reduced for H3K4me3. This analysis not only showed that there was a much higher proportion of sites displaying decreases than increases in H3K27me3 and H3K9me3 for the transition from RBLs to LCLs, but also that these changes affect at many genomic sites. Gene ontology analysis of the gene lists corresponding to the reductions in H3K27me3 and H3K9me3 revealed significant enrichment of categories related to cell division, positive regulation of cell proliferation and apoptosis for H3K27me3 and regulation of transcription and apoptosis for H3K9me3 (Figure 2A). We then looked at the identity of the genes undergoing a decrease in H3K27me3 and H3K9me3. We found that the two modifications were most substantially affecting at the promoters of large classes of transcription factors. For instance, the majority of the *HOX* cluster genes underwent a decrease in H3K27me3 (Figure 2B, top) and most of the zinc finger (*ZNF*) genes and coiled-coil domain-containing (*CCDC*) genes showed a decrease in H3K9me3 (Figure 2B, bottom). This result is coincident with the type of marks that characterizes each of the genes. *HOX* genes are characterized by the presence of H3K27me3, and this mark is maintained through the methyltransferase EZH2 (27,28). *ZNF* genes are generally enriched in H3K9me3 (28).

H3K27me3 and H3K9me3 are also present in constitutive heterochromatin, associated with a variety of repetitive elements including pericentromeric repeats, such as Sat2 (29,30), and subtelomeric sequences like NLB2 and D4Z4 (30). In the case of H4K20me3, this is the most characteristic mark in this type of heterochromatin (2,31). Using ChIP assays, we investigated the association of the three marks with these repetitive elements. Comparison of H4K20me3 in these repeats between RBLs and LCLs revealed an enrichment of this mark for RBLs in these repeats and a marked reduction in LCLs (Figure 2C), whereas H4K20me3 was absent and displayed no significant differences in euchromatic control genes like GAPDH (Figure 2A). These results reinforced the notion that changes in constitutive heterochromatin occur during the EBV-mediated transformation of cells. We observed a similar change for H3K9me3 when comparing RBLs and LCLs (Figure 2C). However, for H3K27me3 we observed enrichment in this mark for Sat2 and NLB2 repeats, consistent with the notion that this mark is also present in constitutive heterochromatin (2), but this mark did not decrease in the transformation to LCLs (Figure 2C).

A reduction of the levels of H3K27me3 and H3K9me3 is likely to be linked to increased expression at the corresponding genomic sites. We tested changes in expression in association with genomic sites undergoing a significant reduction in these heterochromatic marks. To this end, we also used the expression data obtained from the comparison of RBLs and B lymphoblasts (GSE30916) (17). We found that most of the genes undergoing loss of H3K27me3 also had increased expression (Supplementary Table S4 and Figure 2D) (PCC = –0.027). This correlation was not significant for genes undergoing a decrease in H3K9me3 (PCC = 0.067) (Supplementary Figure S4A). The list of genes with an inverse correlation between change in H3K27me3 and expression included several involved in cell cycle regulation and differentiation, such as *CDKN3*, *CDKN2C*, *NFIB* and *SOCS2* (see original ChIP-seq profiles and expression data in Supplementary Figure S4B). Quantitative RT-PCR analysis confirmed the increase in the expression levels of these genes between RBLs and LCLs (Figure 2E). These results suggest that the global decrease in H3K27me3 could have an effect in the upregulation of genes that participate in the acquisition of the transformed phenotype.

### Increased endonuclease accessibility during RBL to LCL conversion

During transformation of resting to proliferating B cells the size of cell nuclei increases suggesting the occurrence of global decondensation. Also, an overall decrease in these histone heterochromatin marks may be associated with increased accessibility to endonucleases. To investigate this possibility, we first compared the DNase-seq profiles of RBLs and LCLs (data from the ENCODE database; analysis described in detail in Materials and Methods section). Comparison of the data corresponding to RBLs and LCLs revealed that 71 349 genomic sites had greater accessibility, whereas only 12 656 showed decreased accessibility (Supplementary Figure S4C). This predominance of higher accessibility is also compatible with the increase in nucleus size and a global gene upregulation. We also tested changes in expression associated with genomic sites that undergo increased accessibility. This analysis led to the identification of 7361 genes undergoing an increase of expression (Supplementary Table S5 and Supplementary Figure S4D). Among the genes that show increased accessibility and higher expression levels we identified a number of genes that are relevant to the cell-growth checkpoints and signal transduction mechanisms. This includes genes like regulator of G-protein signaling *RGS1*, several cytokines *(IL1A*, *IL-4I1*, *IL32*, *CCL3*, *CCR8*, *CCL17*, *CCL22*, *CCL23*, *CCL25*, etc.), various interferon-related genes (*IFNA2*, *IFI44L*, *IFITM1*, *IFIT3*, *IFIH1*, *IRF4*, *IFI27L1*, etc.) as well as well-known EBV-induced genes like *EBI3.*

As indicated above, it is generally assumed that heterochromatic histone marks are associated with a more condensed conformation refractory to endonuclease digestion. To explore the potential relationship between decrease in histone heterochromatic marks and increased endonuclease accessibility, we first combined the DNase accessibility data with genomic sites for differential binding of H3K27me3 and H3K9me3 and found that the overlap was not significant since only a small proportion of sites were actually undergoing both events simultaneously (Supplementary Figure S4E).

**Figure 3.**
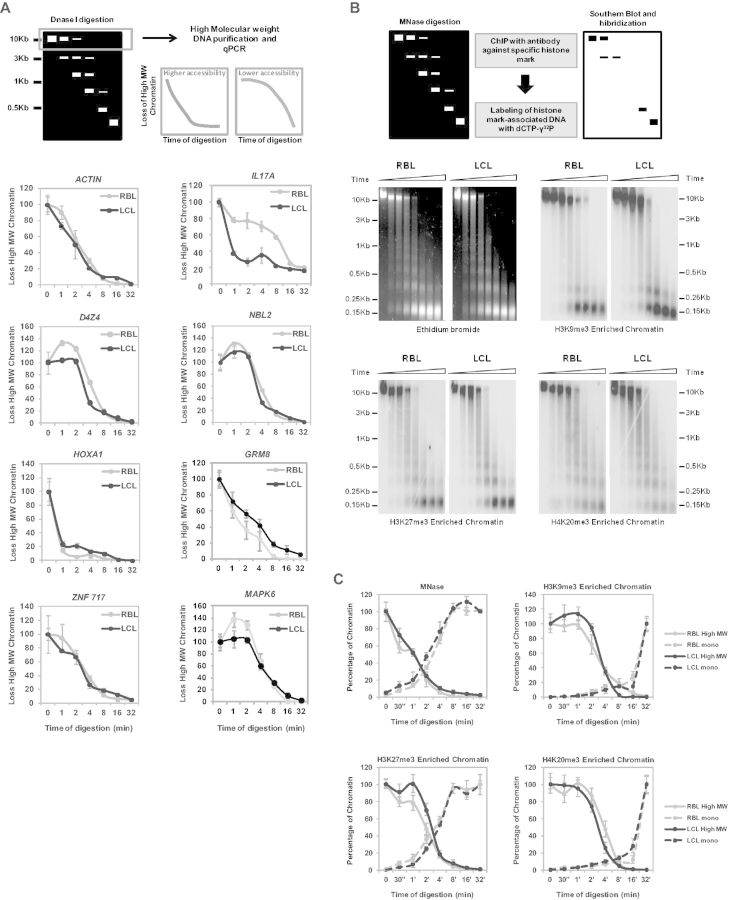
Independence of loss of heterochromatic marks and increased in accessibility during transformation of RBLs to LCLs. (**A**) Top panel, scheme depicting the flowchart of the DNaseI-qPCR analysis. Bottom panel, high molecular weight fragments in a time course DNaseI digestion of RBLs and LCLs. *ACTIN* and *IL17A* are used as negative and positive controls, respectively, of genes with equal or differential accessibility between RBL and LCL. Specific sequences that undergo a decrease in H4K20me3 and H3K9me3 (D4Z4 and NBL2 repeats), H3K27me3 (*HOXA1*, *GMR8*) and H3K9me3 (*ZNF717*, *MAPK6*) were analysed. (**B**) Top panel, scheme depicting the flowchart of the Southern blot-ChIP based analysis of MNase-digested samples. DNA from CHIP experiments with H3K9me3, H3K27me3 and H4K20me3 material are shown. (**C**) Quantitation of Southern blot analysis. Scans of the large DNA fragments and mononucleosomal-size fragments over time are shown.

We then investigated the accessibility at specific sequences that undergo a decrease in H4K20me3 (D4Z4, Sat2 and NBL2 repeats), H3K27me3 (*HOXA1*, *GMR8*) and H3K9me3 (*ZNF717*, *MAPK6*) using DNaseI digestion followed by amplification by quantitative PCR with primers for these sequences. We cut the DNase-resistant fraction (size range ≥5000 bp, see top panel of Figure 3A) at different time points and amplified all the above repetitive elements and other sequences. This strategy allowed us monitoring the dynamics of digestion providing an estimation of accessibility to DNase. In these experiments we used *ACTIN* and *IL17A* as negative and positive controls, respectively, of genes with equal or differential accessibility between RBL and LCL, from the analysis of the DNase-seq data described above. *ACTIN* was found to be rapidly digested in both RBL and LCL, whereas *IL17A* is slowly digested in RBLs but rapidly digested in LCLs. Analysis of these sequences also confirmed the validity of our analysis. In the case of subtelomeric repeats (D4Z4) non-satellite repeats we observed for RBLs an increase in these sequences at early digestions points with respect to initial time (Figure 3A). This is consistent with the higher resistance to digestion of these regions, which are highly compact, that causes a proportional enrichment. In LCLs, we observed a lower enrichment and a higher digestion rate for D4Z4 repeats, compatible with the reduction in this type of constitutive heterochromatin in proliferating cells. However, in other cases (NBL2, *HOXA1*, *GMR8*, *ZNF717*) we did not observe significant differences in the dynamics of digestion between RBLs and LCLs (Figure 3A), indicating that these sequences that display differential enrichment for H4K20me3, H3K27me3 and H3K9me3 were similar in their accessibility to DNase I between these two situations. Interestingly, in other sequences displaying a slow DNase I digestion dynamics in RBLs (*MAPK6*, with a loss of H3K9me3 in LCLs) we also observed increased accessibility in LCLs (Figure 3A, bottom).

**Figure 4.**
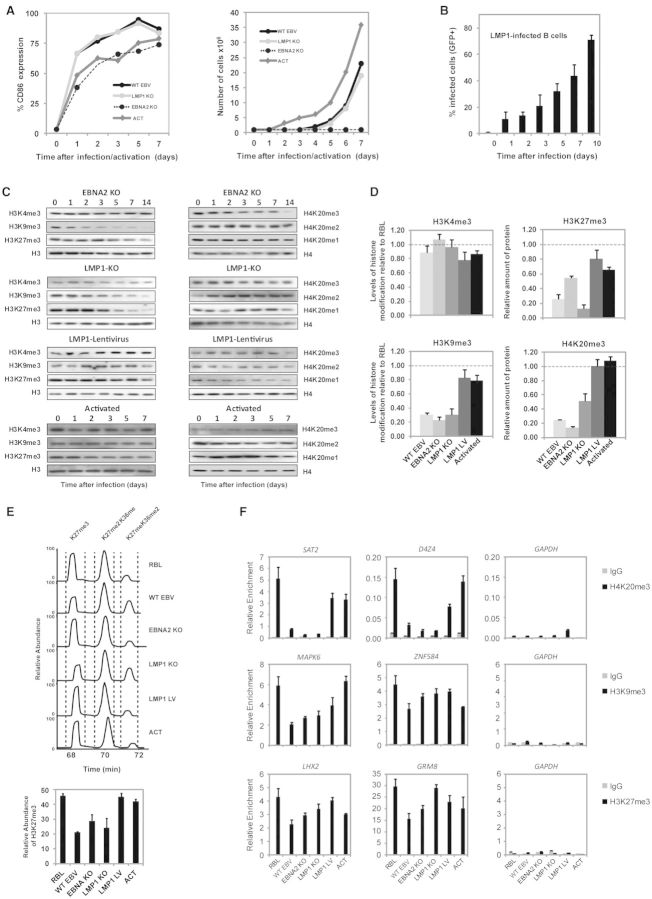
Comparison of histone modification changes during transformation of RBLs with mutant forms of EBV and IL-4/CD40L-stimulated cells. (**A**) Comparison of activation and proliferation levels between B cells infected with wild type EBV (WT EBV), the LMP-1 and EBNA-2 deficient forms of EBV (LMP1 KO and EBNA2 KO), and cells stimulated with IL-4/CD40L (ACT). The left panel shows the proportion of activated cells (left; determined by FACS analysis with CD86) and the right panel the proportion of dividing cells (determined with tritiated thymidine). (**B**) Quantitation of levels of infection with LMP1-expressing lentivirus measured by GFP fluorescence and FACS analysis. (**C**) Analysis by western blot of a time course experiment with antibodies against H3K4me3, H3K9me3, H3K27me3, total H3, H4K20me3 and total H4 of RBLs infected with wild type EBV, EBV deficient for LMP-1, EBV deficient for EBNA-2, IL-4/CD40L-stimulated B cells and IL-4/CD40L-stimulated B cells infected with LMP-1. (**D**) Densitometric quantitation of western blot data. The average of three independent experiments is represented. Data are presented relative to levels in RBL. (**E**) Top panel, XIC of isobaric H3 peptides from different B-cell populations. Differentially modified peptides were chromatographically separated and individual peaks identified by tandem MS spectra. Relative levels of individual isoforms were quantified using the Genesis peak detection module of the Xcalibur software package (Thermo Fisher Scientific). *X*-axis represents retention time, *y*-axis the intensity of ion currents in the Orbitrap mass spectrometer. Bottom panel, bar chart of H3K27me3 levels relative to the two other modification states of this peptide. Error bars represent the SD of two independent biological replicates. *Abbreviations*: unmod = unmodified peptide, me1 = single mono-methylated lysine. (**F**) Quantitative ChIP assays showing changes in H4K20me3, H3K27me3 and H3K9me3 between RBLs and B cells infected wild type EBV, EBV deficient for LMP-1, EBV deficient for EBNA-2, IL-4/CD40L-stimulated B cells and IL-4/CD40L-stimulated B cells infected with LMP-1 (LMP1 LV). The sequences correspond to promoter gene sequences from the ChIP-seq analysis. GAPDH was used as a control sequence.

Finally, we investigated whether increased accessibility was physically associated with specific changes in the histone methylation profile by combining digestion with MNase coupled to hybridization with DNA probes prepared from ChIP experiments with the three different histone modifications (H3K27me3, H3K9me3 and H4K20me3) (Figure 3B, top panel). This type of experiment enabled us to test the accessibility to MNase in sequences bound to these different heterochromatin marks in RBLs and LCLs. We first compared the MNase sensitivity of RBLs and LCLs (Figure 3B, top left). We observed an identical digestion dynamics of bulk chromatin. We then used H3K27me3-, H3K9me3-, H4K20me3- enriched materials from RBLs and hybridized it against both RBL and LCL MNase-digested chromatin fragments. Comparison between RBLs and LCLs did not show any significant difference in the dynamics of accessibility to MNase digestion of the chromatin for any of the three heterochromatic histone marks (Figure 4C and D). However, we cannot discard that changes are occurring in the proximity of the genomic sites adjacent to these heterochromatic histone marks, however for the following experiments we focused on histone modification changes.

### Loss of H3K27me3, H3K9me3 and H4K20me3 is directly associated with EBV-mediated transformation of B cells and not with the acquisition of proliferation

Infection of RBLs with EBV results in activation to lymphoblasts that are phenotypically similar to those generated by physiological stimulation with CD40L plus IL-4. One important difference is that EBV infection leads to the establishment of permanently growing LCLs, whereas CD40L/IL-4 blasts have finite proliferation lifespans. Our results indicated that decrease in H3K27me3, H3K9me3 and H4K20me3 is already substantial at early time points, when the proliferation rates and activation levels of B cells are not so different between cells infected with EBV or stimulated with CD40L/IL-4. To investigate whether the described global chromatin changes are directly associated with EBV-mediated transformation and are not just a mere effect of B cell activation or the acquisition of a proliferative phenotype, we focused on the acquisition of changes in H3K27me3, H3K9me3 and H4K20me3 in B cells activated/stimulated with IL-4 and CD40L. Under our conditions we observed similar levels of cell activation in B cells activated with CD40L/IL-4 when compared to EBV-infected B cells, measured by cytometry analysis of the CD86 surface marker (Figure 4A), and higher proliferation rates for CD40L/IL-4-activated cells, measured by tritiated thymidine incorporation (Figure 4A). However, when looking at the levels of H3K9me3 and H4K20me3 over time we did not observe any decrease in B cells stimulated with CD40L/IL-4, in contrast with our observations for EBV-stimulated cells (Figure 4C, bottom). We only observed moderate changes for H3K27me3 (Figure 4C and D), but much lower than those observed for B cells infected with EBV. These results suggest that differences between these two related mechanisms are relevant in the acquisition of changes in these histone modifications.

In parallel, we stimulated B cells with two different recombinant forms of EBV where the EBNA-2 (32) and LMP-1 (9) genes, the two major EBV-encoded proteins, are deleted. Under these conditions, cells undergo a few divisions but do not acquire the capability of unlimited growth. Analysis of the activation levels for these EBV forms were similar to the levels observed for wild type EBV or cells activated with CD40L/IL-4 (Figure 4A), however B cells infected with EBNA-2-deficient EBV had a much lower proliferation rate than wild type EBV (Figure 4A). Interestingly, we found that B cells infected with any of these two EBV recombinant forms underwent a decrease in H3K27me3, H3K9me3 and H4K20me3 similar to that seen in wild type EBV, regardless of their proliferation rates (Figure 4C and D). These results were independently confirmed by mass spectrometry analysis, which also showed that both forms of EBV (deficient for EBNA-2 or LMP-1) resulted in reduced H3K27me3 levels, albeit to a lesser degree than wild type EBV. In contrast, we did not observe a change in H3K27me3 when B cells were activated with CD40L/IL-4 (Figure 4E). All together, this set of data indicates that the changes observed for these marks in association with EBV infection are not related with the acquisition of the ability to proliferate but it is a direct effect of the transformation mediated through EBV. We also tested the ability of LMP-1 alone to influence on the levels of these histone marks. We chose LMP-1 because its autonomous potential of growth-transforming cells (33). To this end we infected cells previously activated with CD40L/IL-4 with a lentiviral construct expressing LMP-1. We obtained efficient levels of infection with LMP-1 during the course of the first week as quantitated by measuring the incorporation of GFP by flow cytometry (Figure 4B). In this case, we obtained similar results to those in cells activated by CD40L/IL-4 alone, i.e. absence of global changes in heterochromatic marks (Figure 4C and D) with the exception of a moderate reduction in H3K27me3. Overall, these results are in line with the observations by others, where full transformation of B cells by EBV cannot be attributed to a single viral oncogene and rather, the cooperative functions of multiple viral genes are required.

We also compared the acquisition of changes in these marks between wild type and defective EBV forms and CD40L/IL-4-stimulated cells at the gene-specific level by performing ChIP assays. We tested changes in H4K20me3, H3K27me3 and H3K9me3 at candidate sequences and genes selected from our comparison of ChIP-seq data. This analysis not only allowed us to perform gene-specific comparisons between the different situations explored above, but also validated the results from the comparison of ChIP-seq data. We observed that both the wild type and two mutant forms of EBV were able to reduce the levels of H4K20me3 at Sat2 and D4Z4 repeats. We also observed that B cells infected with the LMP1-expressing lentivirus had the levels of H4K20me3 reduced in D4Z4 repeats (Figure 4F). For H3K27me3 and H3K9me3 we observed results in line with the ones obtained in the global analysis, however specific sequences revealed different contributions for each of the conditions (Figure 4F).

## DISCUSSION

Our results provide evidence that EBV-mediated transformation of B cells associates with both global decrease in heterochromatin histone post-translational modifications (H3K9me3, H3K27me3 and H4K20me3) and increased chromatin accessibility to endonucleases. These two types of changes affect thousands of genomic sites. Comparison of these results with those obtained by activating and inducing proliferation with T cell-derived mitogens CD40L and IL-4, as well as with EBV mutants, indicates that the process is specific to the changes occurring associated with grow transformation and not by just pure induction of cell proliferation. We have also observed that the two types of chromatin changes, heterochromatin mark levels and chromatin accessibility, do not necessarily overlap, despite the general notion that heterochromatic marks are associated with chromatin compaction.

Our first finding indicates that loss of heterochromatic histone marks is predominant over changes in euchromatic marks. The observed losses for H3K9me3, H3K27me3 and H4K20me3 are not apparent for activating histone modifications like H3K4me3 or the acetylated forms of histones H3 and H4. Decreases in these heterochromatic marks are not only evident when looking at their global levels, but also when analysing genomic sites to which these marks are associated, as evidenced from both candidate sequence analysis and genome-wide analysis. Changes affect pericentromeric and subtelomeric repeats, especially for H4K20me3 and H3K9me3, and thousands of genomic sites in facultative heterochromatin for H3K27me3 and H3K9me3.

Our analysis also revealed an increase in accessibility to endonucleases when comparing RBLs and LCLs. This finding is compatible with the general observation that conversion of RBLs to LCLs results in a significant enlargement of the nucleus. At the molecular level, accessibility to endonucleases is an experimental measurement of loosening of chromatin. Also, changes in accessibility at genomic sites associated with overexpression of several thousand genes. All together, our study is the first description of a predominant change in heterochromatic marks and chromatin accessibility associated with a functional change in expression during cell transformation.

An unexpected observation was the inability to demonstrate in some cases an overlap between the sequences that undergo a decrease in heterochromatic marks and increased accessibility, despite the general notion that these elements (heterochromatic histone marks and chromatin compaction) are linked. There are several potential explanations for this observation. A sudden change in the cell cycle/division status of the cell, including transformation from a quiescent to proliferative status in aberrant context, may result in inefficient maintenance of the epigenetic marks. We have also observed a similar behavior when focusing on DNA methylation during the conversion of resting to proliferating B cells (34), that is decreased in genes in euchromatic regions, particularly those regulated by B cell specific factors. In this context of inefficient maintenance, different elements of the epigenetic and nuclear organization machinery that are normally coupled may find their links loosened. A study has recently shown independence between chromatin compaction and histone modifications, in cells deficient for DNA methyltransferases (35). However, we cannot discard that there is increased accessibility in regions close (upstream or downstream) to those undergoing these changes in histone modifications and therefore further analysis will be required. In the context of this study, we therefore focused our attention in heterochromatic histone modifications as their changes were more robustly associated with the growth transformation process.

One of the questions arising from these results is whether changes at these marks occur concomitantly with cells acquiring the ability to proliferate, or if an initial change is directly responsible for the deposition or removal of these marks. Recent evidence has shown that increased and decreased activity of enzymes controlling H3K27me3 contributes to carcinogenesis. This includes both EZH2 (26), KDM6B/JMJD3 (25) and UTX. For instance, JMJD3 (the main H3K27me3 demethylase) has been reported to be induced by EBV (25). In addition, other Polycomb group factors are regulated by EBV proteins. For instance, Bmi1 is upregulated by LMP-1 (36). Our analysis on the global levels of enzymes responsible for the maintenance of H3K27me3, H3K9me3 and H4K20me3 revealed certain trends in expression changes of histone methyltransferases (EZH2, for H3K27; SUV39H1 and SUV39H2 for H3K9; and SUV420H1 and SUV420H2 for H4K20) compatible with the observed changes in those histone heterochromatic marks. However, other enzymes did no correlate and further functional dissection will be required to determine whether global changes in these enzymes are related to changes in these histone marks during transformation of B cells.

To address the possibility that the observed changes associated with EBV-mediated transformation of B cells are mainly driven by the acquired ability of cells to proliferate, we compared the results with those obtained by stimulating B cells with IL-4/CD40L. Both EBV-mediated transformation and CD40L/IL-4-activation share cellular pathways (3). In this case, we observed that IL-4/CD40L-mediated activation of B cells that results in active proliferation does not lead to a loss in heterochromatic marks.

Following EBV infection of RBLs *in vitro*, EBNA-2 drives B cell transformation into LCLs by acting as a potent transcriptional activator of both viral and cell genes which in turn will then cause survival and proliferation of the infected cells. Among the viral EBNA-2-targets, LMP-1 is the major EBV oncogene and is required for initiation of primary B cell transformation *in vitro* (8) and also for maintenance of continuous proliferation of EBV-LCLs (9). Interestingly, LMP-1 and EBNA-2 deficient EBV, which are less efficient in stimulating proliferation (particularly EBNA-2 deficient EBV viral particles) still induced the loss heterochromatin marks. This dissection allowed us to separate the effects of proliferation from the direct influence of the EBV infection in transforming B cells. On the other hand, the sole infection with LMP-1 did have only moderate effects in some of these marks, indicating its participation in the acquisition of histone heterochromatin changes but also the need of the cooperative function of additional viral genes, in agreement with the results by others. Recent data have shown a prominent role of other EBV proteins in inducing chromatin changes during transformation. For instance, EBNA-1 mediates recruitment of a histone H2B deubiquitylating complex to the EBV latent origin of DNA replication (37). EBNA-1 has also been shown to interact with multiple nuclear proteins (38) and can promote large-scale chromatin decondensation mediated by a bipartite Gly-Arg domain of this protein that resembles the AT-hook of HMGA (39). Similar effects in chromatin accessibility or induction of histone modification changes have been observed in the homologs encoded by other gamma herpesviruses, for example LANA-1 in Human Herpes Virus 8 (40) or others (41). In this sense, it will be important to test the role of EBNA-1 and other EBV proteins in the acquisition of the chromatin changes described in this study.

As explained above, quiescent differentiated cells have a restricted transcription program, in which only a limited set of genes (ubiquitously expressed and cell type specific genes) are expressed. This progressive restriction of the transcription program, as well as the increase in both facultative and constitutive heterochromatin marks (2,42), is well-known to occur during differentiation. EBV-mediated transformation could therefore be viewed as an inverse process to that occurring during differentiation, and some of the mechanisms involved are likely to act in an opposite direction to those driving differentiation. In this sense, our data fit very well with this notion, since we found that the decrease in some of these heterochromatin marks, specifically H3K27me3, is associated with an increase in expression. However, to the best of our knowledge, this is the first report of such a connection being established. Our observation of decreased levels of H4K20me3 are also compatible with the general finding that loss of this mark is a common hallmark of human cancer (30). Other studies have also shown decreased H3K9me3 and H3K27me3 levels in different cancer types (43–45). In this sense, our findings suggest that B cell transformation-associated loss of H4K20me3 and other heterochromatin histone marks might represent an early step in lymphomagenesis, or more in general, perhaps in carcinogenesis.

Our study has led to identification of a novel phenomenon implicating global chromatin changes related to EBV-mediated transformation of B cells. Our analysis of a related mechanism stimulating B cell proliferation, together with the analysis of the effects of recombinant EBV forms, highlights the association of these changes with growth transformation but not with the pure acquisition of proliferation capabilities.

## SUPPLEMENTARY DATA

Supplementary Data are available at NAR Online.

## FUNDING

Spanish Ministry of Economy and Competitiveness (MINECO) [SAF2011-29635]; Fundación Ramón Areces [CIVP16A1834]; and Catalan Agency for Management of University and Research Grants (AGAUR) [2009SGR184]; Work in A.I.’s lab was funded by the European Union [EpiGeneSys 257082]. Funding for open access charge: MINECO [SAF2011-29635].

*Conflict of interest statement.* None declared.

## Supplementary Material

Supplementary Data
