# Supplementary material for: Epstein–Barr virus-mediated transformation of B cells induces global chromatin changes independent to the acquisition of proliferation
Source: Nucleic Acids Res. 2013 Oct 3;42(1):249–63. doi: 10.1093/nar/gkt886 (PMC3874198; doi:10.1093/nar/gkt886)
Supplement: Supplementary Data [file supp_42_1_249__index.html]

Supplementary Data 

# Epstein–Barr virus-mediated transformation of B cells induces global chromatin changes independent to the acquisition of proliferation

## Supplementary Data

files

**Files in this Data Supplement:**

- Supplementary Data - pdf file
- Supplemental Table 1
- Supplemental Table 2
- Supplemental Table 4
- Supplemental Table 5
- Supplemental Table 3
