# Supplementary material for: Epstein–Barr virus-mediated transformation of B cells induces global chromatin changes independent to the acquisition of proliferation
Source: Nucleic Acids Res. 2013 Oct 3;42(1):249–63. doi: 10.1093/nar/gkt886 (PMC3874198; doi:10.1093/nar/gkt886)

Supplementary Figure 1. Increase of the nuclear size. (A) DAPI staining (red) comparing nuclear size of one RBL with one LCL. (B) Dot plot showing the differences in nuclear diameter between RBLs and LCLs in a total of 50 cell of each.

Supplementary Figure 2. (A) Analysis by western blot of a time course experiment with antibodies against monoacetylated forms of specific histone H4 residues K5, K8, K12 and K16. An antibody against the C-terminus of H4 is used as a control (B) Densitometric analysis comparing the levels in RBLs and LCLs (at 14 d) of H4K5Ac, H4K8Ac, H4K12Ac and H4K16Ac.

Supplementary Figure 3. Heatmap showing the comparison of the expression levels between RBLs and LCLs for a list of histone methyltransferases and histone demethylases specific to H3K27me, H3K9me3 and H4K20me3. Histone demethylases are marked with a green arrow and histone methyltransferases with a red arrow. The heatmap includes all the genes displaying significant expression changes (with $FC \geq 2$ or $FC \leq 0.5$; $p \leq 0.01$). A scale is shown at the bottom, whereby log2 expression values ranges from -1 (less expressed, blue) to 1 (more expressed, red) are shown.

Supplementary Figure 4. (A) Analysis of expression changes for genomic sites undergoing a decrease in H3K9me3. All genes displaying a decrease in H3K9me3 enrichment in LCLs with respect to RBLs (blue bars) are shown including their corresponding change in expression (red bars), both in log2 scale. (B) Examples of CHIP-seq profiles corresponding to RBLs and LCLs for genes with an inverse correlation between change in H3K27me3 and gene expression. Expression data comparison between RBLs and LCLs from the array (GSE30916) for these genes is also shown (C) Comparison of the DNase-seq data between RBLs and LCLs. (D) Analysis of expression changes for genomic sites undergoing an increase in accessibility to DNase is shown. All genes displaying an increase in DNase

accessibility in LCLs with respect to RBLs (blue bars) are shown including their corresponding change in expression (red bars), both in log2 scale. (E) Venn diagrams showing the overlap between different events taking place during the transformation of RBLs to LCLs: H3K9me3 loss and increased DNaseI accessibility, H3K27me3 loss and increased DNaseI accessibility, genes undergoing DNA hypomethylation (36) and H3K9me3 loss, genes undergoing DNA hypomethylation and H3K27me3 loss.

Supp. Figure 1

A

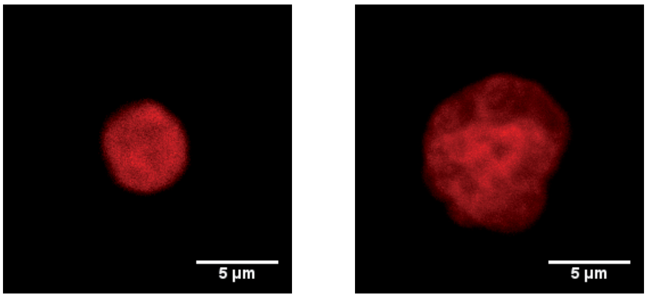

B

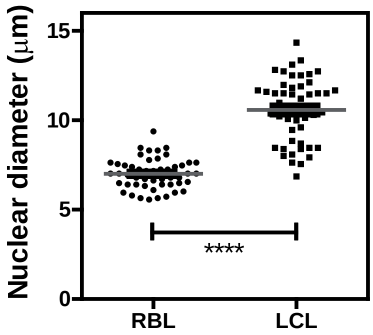

Supp. Figure 2

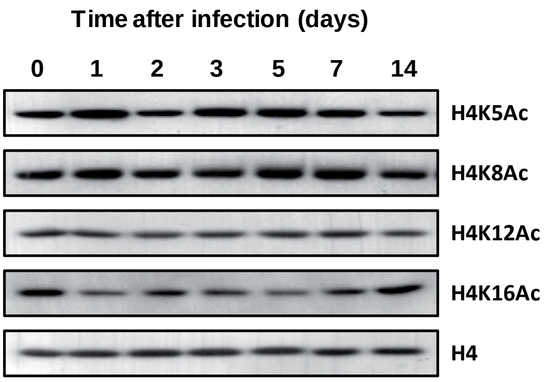

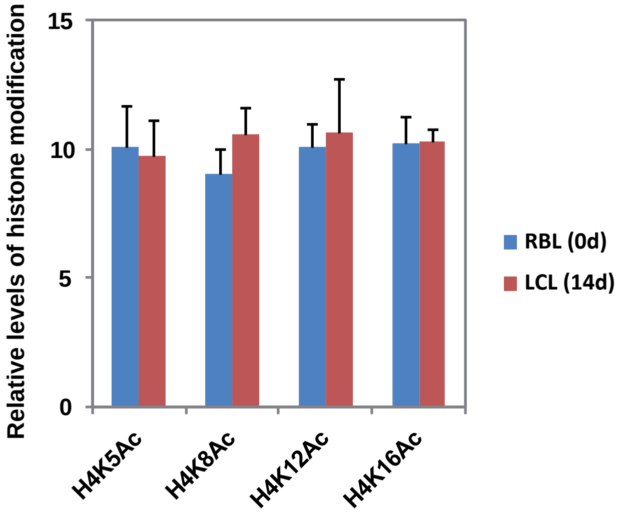

Supp. Figure 3

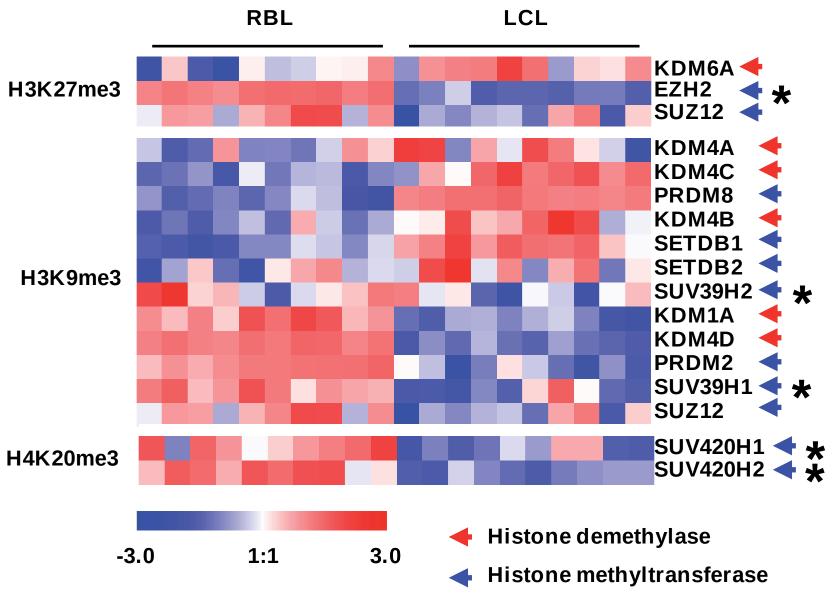

Supp. Figure 4

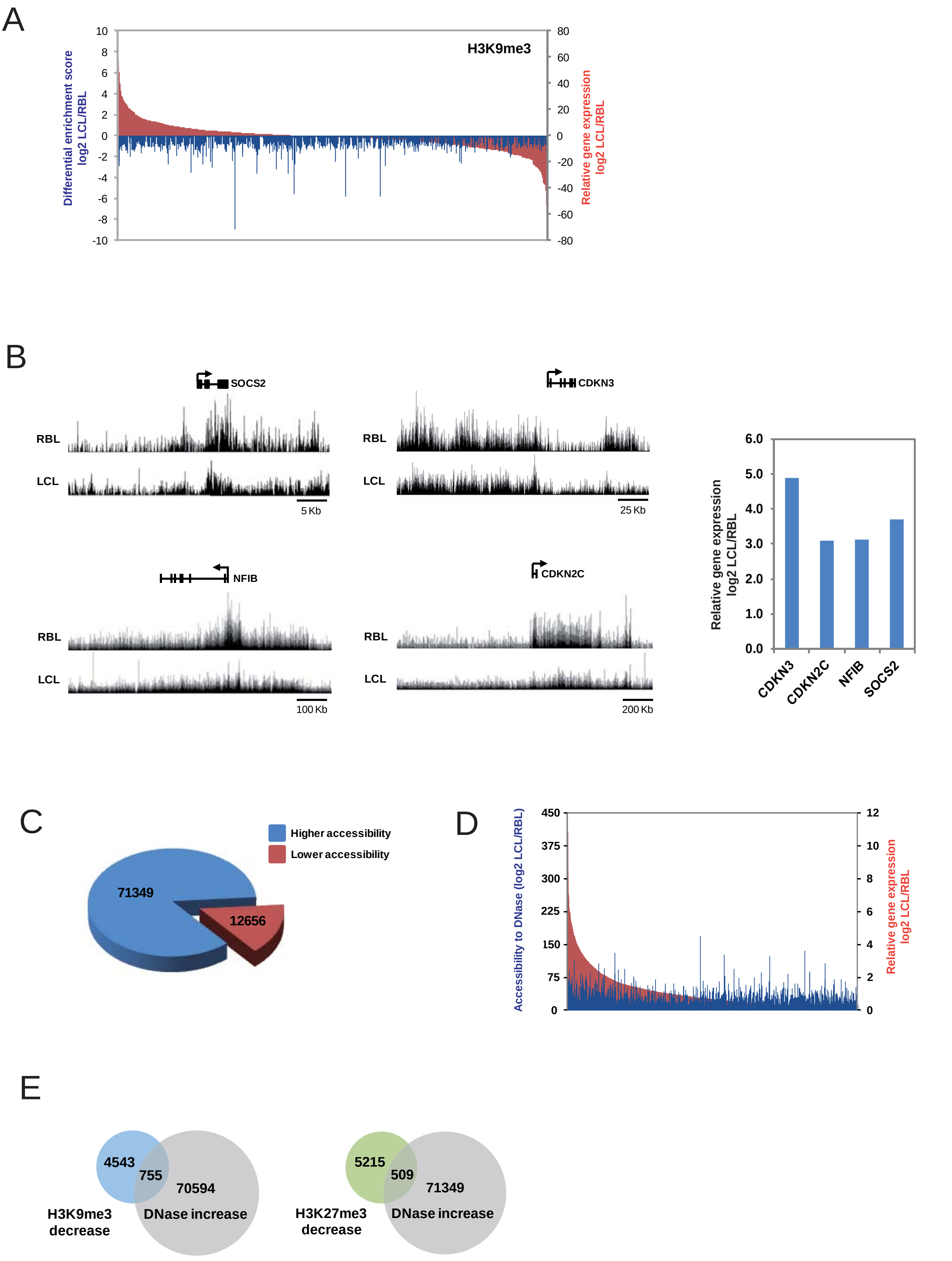

Supplement: Supplementary Data [file supp_gkt886_nar-02187-v-2013-File006.pdf]
