# Supplementary material for: Epstein–Barr virus-mediated transformation of B cells induces global chromatin changes independent to the acquisition of proliferation
Source: Nucleic Acids Res. 2013 Oct 3;42(1):249–63. doi: 10.1093/nar/gkt886 (PMC3874198; doi:10.1093/nar/gkt886)
Supplement: Supplementary Data [file supp_gkt886_Supplementary_Table_1.docx]

**Supplementary Table 1. Primer sequences**

| **GENE** | **TYPE** | **FORWARD** | **REVERSE** |
| --- | --- | --- | --- |
| *SAT2* | Genomic analysis/ChIP | TGAATGGAATCGTCATCGAA | CCATTCGATAATTCCGCTTG |
| *D4Z4* | Genomic analysis/ChIP | CTCAGCGAGGAAGAATACCG | ACCGGGCCTAGACCTAGAAG |
| *NBL2* | Genomic analysis/ChIP | TCCCACAGCAGTTGGTGTTA | TTGGCAGAAACCTCTTTGCT |
| *GAPDH* | Genomic analysis/ChIP | CAATGACCCCTTCATTGACC | GGGGGAATACGTGAGGGTAT |
| *HOXA1* | Genomic analysis/ChIP | GGGTGTCCTACTCCCACTCA | CCCACCACTTACGTCTGCTT |
| *ZNF717* | Genomic analysis/ChIP | CAGCGATTGGTGAGTGGAGA | ATTGGTTCTGCAACTCAGGCT |
| *Beta-ACTIN* | Genomic analysis/ChIP | ATCGTCCACCGCAAATGCTTCTA | AGCCATGCCAATCTCATCTTGTT |
| *LHX2* | Genomic analysis/ChIP | GTTCCAGGCATTTTACATCCA | TAATGGGCGACAAGAAGCTC |
| *MAPK6* | Genomic analysis/ChIP | *ACCGAGGCTAGAACAAAGC* | CAGAAGGAAGCATCCAGGA |
| *ZNF584* | Genomic analysis/ChIP | *GTGCAGGCAAGGTAGGTGA* | TTCCTTGTGGATGTGGTCAA |
| *GRM8* | Genomic analysis/ChIP | TGTACATCGTGCCCTGAAGA | CCGTCTGACATTCACATTGG |
| *IL17A* | Genomic analysis/ChIP | GGCTGGAAGAGCATTCACT | GCTGCTTGCTTTCAGAATCC |
| *SOCS2* | Expression analysis | GAGCTCGGTCAGACAGGATG | TTCCTTCTGGTGCCTCTTTT |
| *CDKN3* | Expression analysis | CGGTTTATGTGCTCTTCCAG | CCACAGCTCTTTAGTTCTTCTGT |
| *RPL38* | Expression analysis |  |  |
