# Supplementary material for: Epstein–Barr virus-mediated transformation of B cells induces global chromatin changes independent to the acquisition of proliferation
Source: Nucleic Acids Res. 2013 Oct 3;42(1):249–63. doi: 10.1093/nar/gkt886 (PMC3874198; doi:10.1093/nar/gkt886)
Supplement: Supplementary Data [file supp_gkt886_Supplementary_Table_2.doc]

**Supplementary Table 2.** Public ChIP-seq Data sources

| **Histone marks** | **Cell type** | **Data source** |
| --- | --- | --- |
| H3K4me3 | CD19  (RBL) | GEO accession GSE19465  [http://www.ncbi.nlm.nih.gov/geo/query/acc.cgi?acc=GSE19465]  **ChIP:**  GSM537622  GSM537632  **Input:**  GSM537620  <http://www.ncbi.nlm.nih.gov/geo/query/acc.cgi?acc=GSM537620> |
| H3K4me3 | GM12878 (LCL) | ENCODE (Broad Institute) [<http://hgdownload.cse.ucsc.edu/goldenPath/hg19/encodeDCC/wgEncodeBroadHistone>]  **ChIP:**  http://hgdownload.cse.ucsc.edu/goldenPath/hg19/encodeDCC/wgEncodeBroadHistone/wgEncodeBroadHistoneGm12878H3k4me3StdAlnRep1.bam  <http://hgdownload.cse.ucsc.edu/goldenPath/hg19/encodeDCC/wgEncodeBroadHistone/wgEncodeBroadHistoneGm12878H3k04me3StdAlnRep2V2.bam>  **Input:**  http://hgdownload.cse.ucsc.edu/goldenPath/hg19/encodeDCC/wgEncodeBroadHistone/wgEncodeBroadHistoneGm12878ControlStdAlnRep1.bam  http://hgdownload.cse.ucsc.edu/goldenPath/hg19/encodeDCC/wgEncodeBroadHistone/wgEncodeBroadHistoneGm12878ControlStdAlnRep2.bam |
|  |  |  |
| H3K27me3 | CD19  (RBL) | GEO accession GSE19465:  **ChIP:**  GSM537634  GSM537638  **Input:**  GSM537620 |
|  |  |  |
| H3K27me3 | GM12878 (LCL) | ENCODE (Broad Institute)  **ChIP:**  http://hgdownload.cse.ucsc.edu/goldenPath/hg19/encodeDCC/wgEncodeBroadHistone/wgEncodeBroadHistoneGm12878H3k27me3StdAlnRep1.bam  http://hgdownload.cse.ucsc.edu/goldenPath/hg19/encodeDCC/wgEncodeBroadHistone/wgEncodeBroadHistoneGm12878H3k27me3StdAlnRep2.bam  http://hgdownload.cse.ucsc.edu/goldenPath/hg19/encodeDCC/wgEncodeBroadHistone/wgEncodeBroadHistoneGm12878H3k27me3StdAlnRep3V2.bam  **Input:**  http://hgdownload.cse.ucsc.edu/goldenPath/hg19/encodeDCC/wgEncodeBroadHistone/wgEncodeBroadHistoneGm12878ControlStdAlnRep1.bam  http://hgdownload.cse.ucsc.edu/goldenPath/hg19/encodeDCC/wgEncodeBroadHistone/wgEncodeBroadHistoneGm12878ControlStdAlnRep2.bam |
|  |  |  |
| H3K9me3 | CD19  (RBL) | GEO accession GSE19465  **ChIP:**  GSM537635  GSM537654  **Input:**  GSM537620 |
| H3K9me3 | GM12878 (LCL) | ENCODE (Broad Institute)  **ChIP:**  http://hgdownload-test.cse.ucsc.edu/goldenPath/hg19/encodeDCC/wgEncodeBroadHistone/wgEncodeBroadHistoneGm12878H3k9me3StdAlnRep1.bam  http://hgdownload-test.cse.ucsc.edu/goldenPath/hg19/encodeDCC/wgEncodeBroadHistone/wgEncodeBroadHistoneGm12878H3k9me3StdAlnRep2.bam  **Input:**  http://hgdownload.cse.ucsc.edu/goldenPath/hg19/encodeDCC/wgEncodeBroadHistone/wgEncodeBroadHistoneGm12878ControlStdAlnRep1.bam  http://hgdownload.cse.ucsc.edu/goldenPath/hg19/encodeDCC/wgEncodeBroadHistone/wgEncodeBroadHistoneGm12878ControlStdAlnRep2.bam |
|  |  |  |
| DNase-seq | CD20 RO01778  (RBL) | ENCODE data  http://hgdownload.cse.ucsc.edu/goldenPath/hg19/encodeDCC/wgEncodeUwDnase/wgEncodeUwDnaseCd20ro01778AlnRep1.bam  http://hgdownload.cse.ucsc.edu/goldenPath/hg19/encodeDCC/wgEncodeUwDnase/wgEncodeUwDnaseCd20ro01778AlnRep2.bam |
|  | GM12878 (LCL) | http://hgdownload.cse.ucsc.edu/goldenPath/hg19/encodeDCC/wgEncodeUwDnase/wgEncodeUwDnaseGm12878AlnRep1.bam  http://hgdownload.cse.ucsc.edu/goldenPath/hg19/encodeDCC/wgEncodeUwDnase/wgEncodeUwDnaseGm12878AlnRep2.bam |
